# Dr. James Gregory Mumford

**Published:** 1914-11

**Authors:** 


Dr. James Gregory Mumford, Harvard, 1890, died at the Clifton Springs Sanitarium, October 18, aged 51. He was born in Rochester but was for some years a practitioner of

Boston and a teacher in his alma mater. Two years ago, he assumed the superintendency of the Clifton Springs Sanitarium. He was a noted surgeon, a review of his last work, a masterly systematic treatise having appeared in the October issue.

